# Epigenetic Regulation of Neuroinflammation in Alzheimer’s Disease

**DOI:** 10.3390/cells13010079

**Published:** 2023-12-29

**Authors:** Yajing Ma, Wang Wang, Sufang Liu, Xiaomeng Qiao, Ying Xing, Qingfeng Zhou, Zhijian Zhang

**Affiliations:** 1 College of Biology and Food, Shangqiu Normal University, Shangqiu 476000, China; mayajing90@126.com; 2 Department of Physiology and Neurobiology, School of Basic Medical Sciences, Zhengzhou University, Zhengzhou 450001, China; zzuwwuzz@163.com (W.W.); xingy@zzu.edu.cn (Y.X.); 3 Department of Biomedical Sciences, College of Dentistry, Texas A&M University, Dallas, TX 75246, USA; sufang.liu@tamu.edu; 4 Department of Pathology and Forensic Medicine, School of Basic Medical Sciences, Zhengzhou University, Zhengzhou 450001, China; xiaomeng416520@126.com

**Keywords:** Alzheimer’s disease, epigenetics, inflammation, microglia, DNA methylation, histone modification

## Abstract

Alzheimer’s disease (AD) is a chronic and progressive neurodegenerative disease and clinically manifests with cognitive decline and behavioral disabilities. Over the past years, mounting studies have demonstrated that the inflammatory response plays a key role in the onset and development of AD, and neuroinflammation has been proposed as the third major pathological driving factor of AD, ranking after the two well-known core pathologies, amyloid β (Aβ) deposits and neurofibrillary tangles (NFTs). Epigenetic mechanisms, referring to heritable changes in gene expression independent of DNA sequence alterations, are crucial regulators of neuroinflammation which have emerged as potential therapeutic targets for AD. Upon regulation of transcriptional repression or activation, epigenetic modification profiles are closely involved in inflammatory gene expression and signaling pathways of neuronal differentiation and cognitive function in central nervous system disorders. In this review, we summarize the current knowledge about epigenetic control mechanisms with a focus on DNA and histone modifications involved in the regulation of inflammatory genes and signaling pathways in AD, and the inhibitors under clinical assessment are also discussed.

## 1. Introduction

Alzheimer’s disease (AD) is a chronic and progressive neurodegenerative disease, with most commonly presented symptoms including cognitive impairment, memory loss, spatial dysfunction, psychiatric disorder, etc. AD typically occurs in people over the age of 65 and also shows an alarming increasing occurrence largely due to the aging population. AD is a pathologically multifactorial disease, and many factors are proposed to contribute to the pathogenesis and progression of AD such as amyloid-beta (Aβ) protein deposits and neurofibrillary tangles (NFT) induced by tau protein hyperphosphorylation, in association with synapse loss, neuronal cell death, chronic neuroinflammation, etc., [1,2]. Although amyloid cascade has long been regarded as the most prevalent hypothesis, accumulating evidence suggests that this hypothesis is insufficient in explaining many aspects of AD pathogenesis and by far there remains no effective treatment for AD pathology based on this hypothesis [3,4,5]. Since a number of evidence has accumulated showing that AD risk factors are more closely associated with inflammation response, the inflammation hypothesis has lately gained increased support as an early detrimental event related to the onset and clinical progress of AD [6,7,8]. In the early stages of AD, glial cell-mediated immune response in AD is involved in the clearance of Aβ, but with the progression of the disease, the excessive inflammatory response in turn aggravates the pathological process of AD. Neuroinflammation in the brain has already been proposed to be an additional core pathological change through the whole pathological course [9,10].

Inflammation is a defensive pathological response of body tissues to the external stimulus such as pathogens, damaged cells, irritants, or endogenous signals and forms an important part of non-specific immunity [11,12]. Acute inflammation is an early and immediate response to injury or damage with the main purpose of interference or tissue repair, while chronic inflammation ensues if injurious factors persist to cause slow and long-term inflammatory reactions [12]. In chronic inflammation, there are a variety of inflammatory cell infiltrations and cytokine secretion, and the resultant damage is closely related to multiple human chronic diseases such as cancer, diabetes, and CNS disorders. Neuroinflammation is an innate and complex immune response mediated by microglia and astrocytes in the CNS [13]. In the occurrence of brain inflammation, acute inflammation plays a defensive role against nervous injury, infection, and other stimuli, characterized by the activation of immune cells, infiltration of peripheral inflammatory cells, and release of various inflammatory factors [14]. But when the equilibrium between anti-inflammatory and pro-inflammatory signaling is disrupted, as seen in AD, chronic inflammation occurs, which is attributed to activated microglia and the release of diverse cytokines [3,5,15]. Sustained inflammation in the brain is considered to be related not only to neurodegeneration but also exacerbates both Aβ and tau protein pathology, indicating inflammation as a fundamental trigger for AD pathogenesis [5,16]. Despite extensive investigation of inflammatory mechanisms, the inflammation transition between protective and pathological roles remains unclear.

In recent years, epigenetic modification has emerged as a fundamental mediator in many human diseases such as CNS disorder [17,18]. Epigenetic modification refers to heritable changes in gene expression independent of DNA sequence alteration and mainly includes DNA methylation, histone modification, non-coding RNAs, chromatin remodeling, nucleosome positioning, etc., [19]. To date, a growing body of studies has confirmed that the epigenetic mechanism is widely involved in biological processes such as embryonic development, stem cell regulation, cell apoptosis, and organ homeostasis maintenance and is closely related to the occurrence and progression of various human diseases [20,21,22]. Upon regulation of transcriptional repression or activation, epigenetic modifiers are considered regulators of complex mechanisms underlying inflammatory signaling in neuronal differentiation and plasticity, as well as cognition functions in CNS diseases [23,24].

**Figure 1:**
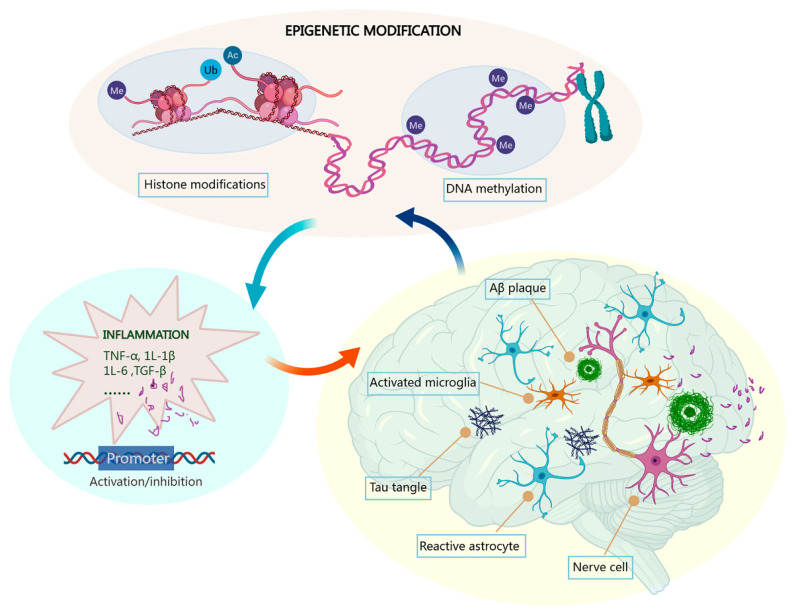
Epigenetic mechanisms via DNA methylation and histone modifications in neuroinflammation in AD. Inflammatory responses mediated by activated microglia play an essential role in initiation and progression of AD. Aberrant epigenetic modifications of gene promoters of cytokines such as TNF-α, IL-1β, and IL-6 promote the inflammatory response of microglia and astrocyte and provoke the formation of pathological Aβ deposits and neurofibrillary tangles, resulting in the development and aggravation of AD. Abbreviations: Me: methyl; Ac: acetyl; Ub: ubiquitin.

A percentage of studies have suggested that epigenetic alteration in brain regions and peripheral lymphocytes could cause inflammatory responses, microglia hyperactivation, and CNS inflammation amplification, resulting in the pathological development of AD (Figure 1) [25,26]. Understanding the epigenetic mechanisms in inflammatory response will provide additional valuable insights for AD pathophysiological processes. The current review focuses on the epigenetic regulation of neuroinflammation in AD pathology, with an emphasis on DNA and histone modifications.

## 2. Retrieval Strategy

The PubMed database and Google Scholar were searched for articles in the literature published from 2000 to the present, especially during the last five years, using a combination of keywords: Alzheimer’s disease, epigenetic, neuroinflammation, DNA methylation, histone modification, microglia.

## 3. Neuroinflammatory Response in AD

Neuroinflammation has long been thought to be a passive function caused by Aβ plaques and neurofibrillary tangles; however, accumulating evidence points to the involvement of inflammation in triggering the neuropathological changes presented in AD [5]. Microglia are the primary phagocytes of the innate immunity in the brain, playing an important role in the maintenance and plasticity of neuronal circuits [27]. In an AD brain, microglia are found in an increased amount in proximity to Aβ plaque, which is hypothesized to clear Aβ deposits via phagocytosis and degradation [28,29]. Additionally, microglia could be activated through its binding to soluble Aβ oligomers and fibrils and then release a variety of pro-inflammatory signal molecules such as cytokines, growth factors, chemotactic factors, and cell adhesion molecules. These cytokines, in turn, promote the amyloid precursor protein production, which results in an increased amount of insoluble Aβ,creating a vicious circle [30]. In addition, the produced complement molecules may participate in the autoimmune reaction in the CNS and further aggravate neuron degeneration and damage. This cascade process is considered to be part of the inflammatory response in AD initiation and progression [31,32,33]. In a recent study, Pacoal et al. for the first time demonstrated that microglial activation is an important driver for the spread of tau tangles over the neocortex in AD. They found that Aβ pathology could promote tau propagation over the neocortex by enhancing the microglial activation, suggesting microglial activation as a key factor to link Aβ pathology and tau spread, proposing that the simultaneous appearance of Aβ protein, tau tangles, and abnormal microglial activation may synergize to drive the occurrence and progression of AD [34]. Additionally, clinical investigation of AD also confirms that cognitive decline and disease aggravation are closely related to acute and chronic systemic inflammation [35].

As an important component of innate immunity, NLRP3 inflammasome is important for immune response and disease occurrence. NLRP3 can recognize pathogen-associated molecular patterns and host-derived danger signal molecules that trigger the NLRP3-dependent immune response [36,37]. NLRP3 can also recruit and activate the pro-inflammatory protease caspase-1 to promote the release of inflammatory factors such as IL-1β and TNF-α, as well as the production of multiple chemokines. Heneka et al. reported that NLRP3 deficiency could attenuate spatial memory impairment and other AD-related pathological symptoms, reduce caspase-1 and IL-1β activation, as well as promote clearance of Aβ peptides [29]. In patients with AD, the pro-inflammatory cytokines in the brain parenchyma are observed to be significantly higher than those of normal people, which may constantly activate the immune system of the CNS to further trigger neuroinflammation and cause AD aggravation [23,35,38].

Nuclear receptor-related factor 1 (Nurr1) is an orphan nuclear receptor with neuroprotective and anti-inflammation properties, playing a role in the development and maturation of midbrain dopamine neurons [39,40,41]. In a very recent mouse model, Yang et al. reported that coexpression of Nurr1 with its co-factor forkhead box protein A2 (Foxa2) using adeno-associated virus serotype 9 (AAV9)-mediated gene delivery in the cranium could efficiently attenuate Aβ-induced neuroinflammation, promote the expression of neurotrophic factors, and improve the cognitive dysfunction of AD, which provides an additional disease-modifying treatment strategy for AD therapy [42]. In addition to being a driving factor for AD pathology, the Aβ protein is also confirmed as an antimicrobial peptide for the innate immune response of the brain [43,44,45]. However, the causal relationship and regulatory mechanism between Aβ-related immune response and AD pathogenesis is not clear. Hur et al. found that pro-inflammatory cytokines are able to induce the generation of interferon-induced transmembrane protein 3 (IFITM3) in neurons and astrocytes, which forms a complex with γ-secretase to thereby promote Aβ generation, providing a new mechanistic insight into inflammation-mediated Aβ production to drive AD pathology and progression [45]. Moreover, their findings elucidate for the first time the relationship between inflammation and AD plaque development and indicate that IFITM3 may serve as a new marker and target for AD diagnosis and treatment.

## 4. Epigenetic Regulation in Inflammation

### 4.1. DNA Modifications in Neuroinflammation

**Figure 2:**
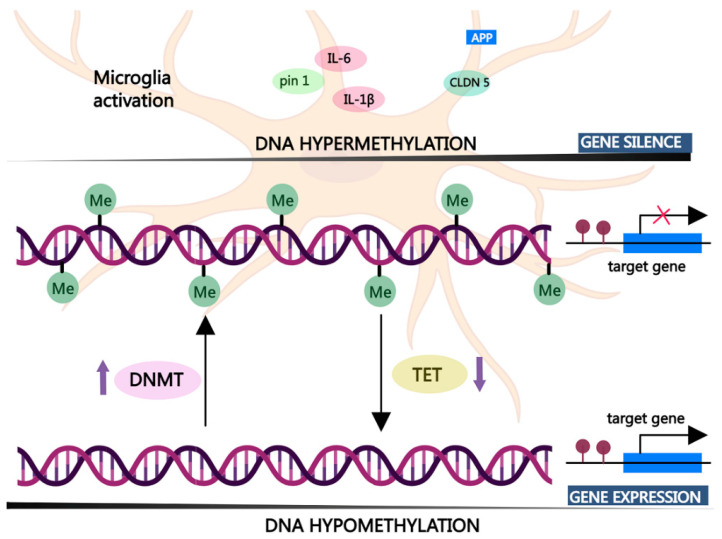
DNA de/methylation status is closely related to the expression of inflammatory cytokines in activated microglia. DNA hypermethylation is associated with gene silencing, while DNA hypomethylation correlates with gene activation. Abbreviations: pin 1: peptidyl-prolyl cis/trans isomerase; CLDN 5: claudin-5; TET: ten-eleven-translocation protein.

DNA methylation is one of the most typical and stable epigenetic marks which plays a fundamental role in regulating gene expression, as well as cell biology and organ formation. Dynamic equilibrium of DNA methylation is essential for the maintenance of normal physiological functions. DNA methylation is generally considered to act as a gene-silencing regulation, and abnormal DNA methylation may lead to aging-related diseases, such as malignances, autoimmune disease, and neurodegenerative disorder [20,46]. DNA methylation catalyzed by DNA methyltransferase (DNMT) mainly occurs at gene promoter sites where the methyl group is covalently added to result in the binding block of transcriptional enzymes and is closely related to inflammatory response and glial reactivity. Generally, DNA hypermethylation is associated with the silencing of gene expression, while hypomethylation may induce the promotion of gene expression (Figure 2) [47]. For instance, decreased methylation of the gene promoter region of APP and APOE occurs in the brain of patients with AD, typically leading to the overexpression of inflammatory molecules further to cause Aβ deposits [48]. Similarly, a reduction in methylation levels also enables the activation of microglia and astrocytes, which potentially causes a vicious cycle of multiple disease pathologies and progression [49]. In addition, aging has been considered as a major risk factor for AD development, and DNA methylation is typically altered with an increase in age. There is a significant increase in PSEN1 expression, which can be attributed to reduced methylation at specific CpG sites, attributing to the buildup of toxic pathogenic Aβ peptides. Hernandez et al. found that the DNA methylation level at the CpG site in the genome of human donors is positively correlated with chronological age, indicating that age-related DNA methylation alterations highly affect gene expression in the brain [50].

The triggering receptor expressed on myeloid cells 2 (TREM2) belonging to the immunoglobulin superfamily is a key receptor expressed on the surface of microglia which regulates the activation and survival of microglia [51,52]. TREM2 is confirmed as an important susceptibility gene to AD, and its expression is positively correlated with AD progression [53,54]. In AD, TREM2 plays a potentially neuroprotective role by inhibiting the microglia-mediated inflammatory reaction [55,56]. A clinical investigation by Ozaki et al. showed that the expression of TREM2 mRNA was increased in peripheral leukocytes of AD, while TREM2 DNA methylation was down-expressed. Their research further presented that TREM2 mRNA expression was negatively correlated with the methylation rate of specific CpG sites in TREM2 intron 1, and therefore the low methylation of CpG sites of TREM2 may serve as an additional marker for AD pathology [57].

#### 4.1.1. DNA Methylation in Neuroinflammation

DNA methyltransferase (DNMTs) are the key enzymes responsible for the DNA methylation process, by which a methyl group is specifically transferred from the methyl donor S-adenosylmethio-nine (SAM) to the 5-position of cytosine to generate 5-methylcytosine [58]. DNMTs play an essential role in dynamic DNA methylation and transcriptional modulation in the genome and also are required for adult memory, learning, and cognition [59,60]. Francesco et al. reported that DNMT1 expression was increased in late-onset AD patients, along with DNA methylation elevation which is positively correlated with the AD risk gene APOE ε4, proposing that global DNA methylation could serve as a reliable marker for AD [61].

DNA methylation plays different roles in the two forms of AD, namely familial AD and sporadic AD [62]. The three major pathogenic genes for familial AD are PSEN1, PSEN2, and APP, while the major gene closely associated with sporadic AD is APOE. The 5’ region of the APP gene is enriched with CpG dinucleotides, and alterations in its methylation levels can influence the expression of APP, thereby affecting the deposition cascade of Aβ protein. Studies have found differential hypomethylation of the APP gene in the brains of AD patients compared to normal controls and significant differences in the methylation levels of the APP gene across different human tissues. However, the methylation patterns of the PSEN1 and PSEN2 genes show no significant differences between AD samples and normal controls [63]. Regarding APOE, research indicates a significant decrease in the DNA methylation levels of APOE in the brain tissues of patients with AD [64]. Hüls et al. conducted a study on the association between brain DNA methylation and cognitive trajectory in AD subjects and found that methylation of CLDN5, a protein-coding gene regulating the permeability of the blood–brain barrier, is associated with cognitive trajectory. And their finding indicated that blood–brain barrier dysfunction induced by abnormal methylation of CLDN5 may play an important role in early cognitive decline in AD [65]. In addition, Pin1 is a propyl-cis-trans isomerase to catalyze the cis-trans isomerization of the tau protein and amyloid precursor protein, and therefore it is associated with the onset and pathology of AD [66,67,68]. Ma et al. found that Pin1 expression was positively correlated with its methylation, and Pin1 methylation elevation was thought to be a risk factor of AD [69]. Mitochondria play an important role in energy metabolism to meet the energetic needs of a living cell, and their dysfunction is tightly related to the pathology of CNS disorders [70,71]. A series of findings showed that DNMTs such as DNMT1 and DNMT3a/3b are observed in mitochondria, referred to as mtDNMTs, and contribute to the methylation of mitochondria in part to regulate the function of mitochondria [71,72,73]. In addition, mitochondrial DNA oxidative damage was found to show early and persistent hypometabolism, and it is proposed that AD oxidative damage contributes to increased rates of mtDNA mutation, as well as retrograde responses of cells to compensate for mitochondrial defects [74,75].

#### 4.1.2. DNA Demethylation in Neuroinflammation

Ten-eleven translocation methylcytosine dioxygenase 1 (TET1) belongs to the TET family that specifically enables the catalytic conversion of 5-mC to 5-hydromethycytosine (5-hmC) to initiate DNA demethylation activation [76,77]. TET1 is associated with memory formation, hippocampal neurogenesis, and cognitive function by regulating DNA methylation and, therefore, gene expression regulation [78]. Knockdown of TET1 can lead to impaired neuronal regeneration, increased inflammatory response, and declined learning and memory ability [79]. Similarly, Tet2 deficiency in the hippocampus also leads to the initiation of inflammatory responses at an early stage, causing further aggravation of AD pathology and cognitive dysfunction particularly [80,81].

5hmC is an intermediate metabolite of the DNA demethylation process and is particularly abundant in the neuronal system [82]. The distribution and intensity of 5hmC is dynamic and region-specific, with particular enrichment in neurons particularly. Pastor et al. developed a high-throughput sequencing of 5hmC-containing DNA to show that it is specifically enriched near the transcriptional start sites of genes that are related to gene transcription and translation [83]. 5hmC exhibits a dual role in both initiating DNA demethylation processes and representing a stable epigenetic marker [84,85]. Dysregulation of 5hmC is closely associated with the pathogenesis and progression of AD [86,87]. The overall expression of 5hmC is found to be evidently reduced in the hippocampus of an aged APPswe/PSEN1 mouse model [80,88]. In the late stages of AD, 5hmC expression is found to be significantly decreased and negatively correlated with increased Aβ deposits. In addition, Shu et al. found that 5hmC expression in the hippocampus could be particularly reduced after the treatment of the Aβ peptide, without changes in other regions such as the cortex and cerebellum. Their findings indicate that differential 5hmC modification plays an important role in gene levels related to neural projection and neurogenesis [88,89]. Moreover, Zhang et al. discovered that TET enzyme activity was decreased during AD progression, which was responsible for 5hmC reduction. However, overexpression of TET catalytic domains to activate TET enzyme activity was found to significantly improve AD pathology such as toxic Aβ peptide clearance and tau hyperphosphorylation reduction, as well as synaptic dysfunction amelioration [90]. Collectively, the dysregulation of 5hmC-mediated demethylation plays a crucial role in the progression of neurodegeneration, and maintaining a regular expression of 5hmC is important to the proliferation and differentiation of neural cells and therefore to ameliorate AD pathology and cognitive impairments.

### 4.2. Histone Modification in Neuroinflammation

Histone is an octamer composed of H2A, H2B, H3, and H4. The amino-terminal tails of histone protruding from core histones are subject to a wide range of chemical modifications such as acetylation, phosphorylation, methylation, ubiquitination, and ADP-ribosylation. These posttranslational modifications are thought to potentially alter the tertiary structure of chromosomes and thereby regulate gene expression in disease initiation and progression [91,92]. For CNS diseases, mounting evidence suggests that histone modifications play a crucial role in diverse biological processes such as neuroinflammation and neuron development in aging and AD models [93,94,95].

#### 4.2.1. Histone Methylation in Neuroinflammation

**Figure 3:**
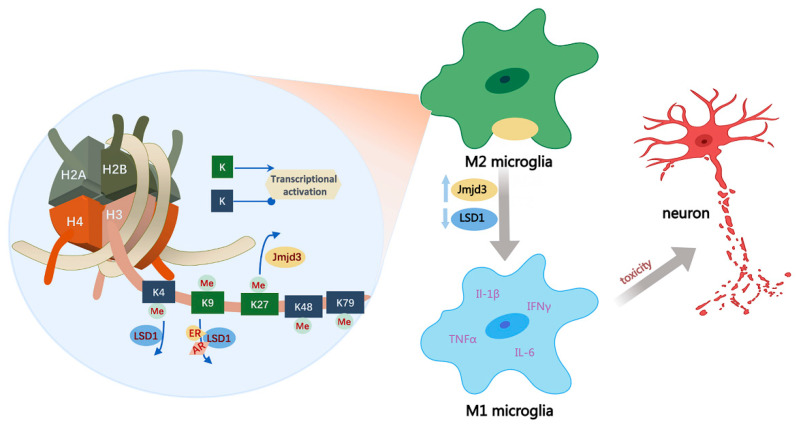
Histone methylation at specific sites causes gene transcriptional activation or repression. Dysregulated histone methylation has a vital role in microglial cell polarization to affect the proinflammatory cytokine production to cause neurotoxicity. Abbreviations: AR, androgen receptor; ER: estrogen receptor; JMJD3: Jumonji domain-containing protein-3.

A number of findings have suggested that histone methylations are closely connected with chromatin remodeling and gene transcription, which are related to inflammation-associated disorders [96]. Histone methylation is dynamically regulated by histone methyltransferase (HMT) and histone demethylase (HDM), which mainly occurs on lysine and serine sites. The effect of histone methylation on gene expression is related to the position and degree of methylation. Multiple lines of evidence show that histone methylation has a critical role in affecting neuronal differentiation and gene expression, while dysregulation of histone methylation is closely related to the pathology of neurodevelopmental diseases [97,98]. For example, methylation of H3K4, H3K48, and H3K79 is associated with transcriptional activation, while H3K9 and H3K27 methylation causes transcriptional downregulation [99,100]. Microglia polarization is closely related to inflammation responses and the release of inflammatory cytokines [101,102]. In M2 microglia, referred to as alternative activation stage, Jmjd3, a specific demethylase of H3K27me3, was confirmed as a crucial regulator for M2 microglia polarization by exerting its demethylase activity [103]. Jmjd3 inhibition could cause the transformation of M2 to the M1 phenotype of microglia and facilitate the release of pro-inflammatory factors such as IL-1β and IL-6, suggesting that Jmjd3 plays a protective role in the CNS (Figure 3) [103].

LSD1, also known as KDM1A, is a histone lysine-specific demethylase which is specifically responsible for removing the mono or dimethylated histone H3 lysine 4 (H3K4), and it is also able to demethylate H3K9me1/2 by complexing with androgen receptors [104]. On the one hand, LSD1 plays a positive role in neural development and neuronal differentiation, and it is constantly required for neuronal progenitor cell maintenance [105,106]. Zhang et al. found that LSD1 was important for neuronal progenitor cells during cortical development by regulating H3K4 methylation of the LSD1-binding site downstream of atrophin 1 [105]. Christopher et al. reported that genetic depletion of LSD1 caused transcriptional alterations in neurodegenerative pathways and activation of stem cell genes in the hippocampus, resulting in paralysis and cognitive deficits [107]. On the other hand, LSD1 functions as a negative regulator in inflammation initiation, showing therapeutic potential in neurodegenerative disorders. LSD1 is reported to be involved in the PKCα-LSD1-NF-κB axis, which is able to stimulate and exacerbate the inflammatory reaction, and pharmacological intervention of LSD1 can ameliorate the systemic inflammatory response [108]. In addition, LSD1 is also implicated in the NF-κB signaling cascade pathway, and LSD1 inhibition was found to be able to reduce the inflammatory cells’ recruitment to tissues by regulating NF-κB signaling to prevent cytokine generation [109].

#### 4.2.2. Histone Acetylation in Neuroinflammation

**Figure 4:**
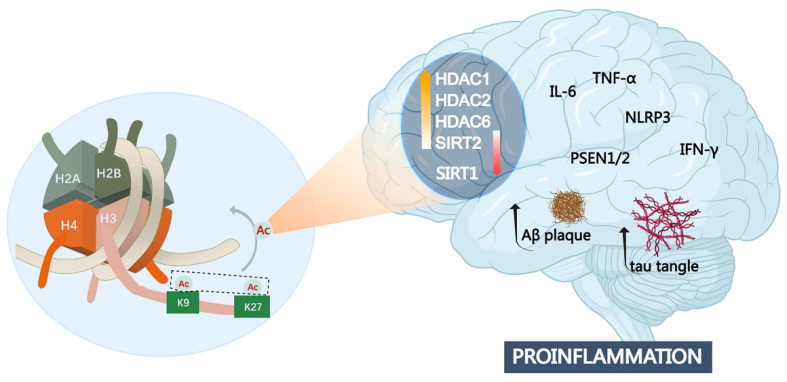
Histone hypoacetylation at both H3K9 and H3K27 sites involved in AD pathogenesis. Aberrant expression of multiple histone deacetylases such as HDAC1, HDAC2, HDAC6, SIRT1, and SIFT2 is closely related to inflammatory response, as well as Aβ deposits and tau pathology. Abbreviations: NLRP3: NOD-like receptor thermal protein domain-associated protein 3; PSEN 1: Presenilin 1.

Histone acetylation modification is one of the most common epigenetic regulations and is dynamically controlled by histone deacetylases (HDACs) and histone acetylases (HATs). Histone acetylation can cause loose packing of chromatin to enable access for DNA binding proteins and further activate gene expression [110]. Studies have shown that histone acetylation in promoter and enhancer regions is an essential prerequisite of target gene activation, and acetylation of H3K27 is a critical marker of transcriptionally active promoters and enhancers [111]. Marzi et al. reported that acetylation modification of the entorhinal cortex in the brain tissue of AD patients revealed more than 4000 differential acetylation modification sites pertaining to genes such as APP, PSEN1/2, and MAPT [112]. Abnormal histone acetylation has been proposed as a common mechanism of epigenetic dysregulation in the pathogenesis of AD (Figure 4) [98,113,114].

In the typical pathological changes of AD, histone acetylation dysregulation not only impacts the expression of memory-related genes but also contributes to the dysregulation of multiple signaling pathways such as cell differentiation, inflammatory response, and vascular and neuronal remodeling. An integrated multiomics analysis by Natio et al. presented that histone acetyltransferase-related genes were increased in expression in the brains of patients with AD. Further proteomic analysis showed that both H3K27ac and H3K9ac were specifically enriched in AD to contribute to the aggravation of neurodegeneration. Their studies disclosed that the acetylation of H3K27 and H3K9 has an important role in driving AD pathogenesis by regulating transcription and the chromatin–gene feedback pathway, providing novel insights for AD initiation and potent intervention [115]. Fang et al. found that histone acetylation dysregulation was a key mechanism of autophagy deficiency and NLRP3 inflammasome activation associated with cognitive impairment in a sevoflurane-induced animal model. The restoration of histone acetylation of H3 and H4 by SAHA, a histone deacetylase inhibitor, was able to activate autophagy and attenuate NLRP3 inflammasome and therefore reduce the excessive inflammatory response to ameliorate cognitive deficiency [116].

In contrast, histone deacetylation leads to chromatin compaction and repression of gene expression. Wang et al. found that exogenous addition of APP in the brain of C57Bl/6J mice could increase the binding of HDAC2 to the Bdnf promoter region, thereby inhibit transcription of the related gene [117]. Compared with normal peers, HDAC6 expression in the cerebral cortex and hippocampus of AD patients increased by 52% and 91%, respectively, and genetic depletion of HDAC6 in APP/PS1 mice had a marked ameliorative effect on the memory impairment of the mice model [118]. In addition, Cook et al. found that HDAC6 inhibition resulted in a significant reduction in tau protein aggregation and further clearance and improved mitochondrial damage induced by Aβ [119,120]. Moreover, different from other HDACs, the level of SIRT1 in the cerebral cortex of AD patients is significantly reduced compared with that of normal samples, which may be associated with Aβ formation as well as tau aggregation in AD brains [121,122]. The deacetylase sirtuin 2 (SIRT2), abundant in the brain, plays a negative role in regulating microglia-mediated inflammation and neurotoxicity. Knock-down of SIRT2 could lead to increased microglia activation associated with pro-inflammatory cytokines, while its overexpression could prevent the activation of microglia [123]. However, SIRT2 inhibition in its activity was proposed in many studies to play an active role in neuroprotection. This discrepancy may be attributed to the fact that genetic depletion and pharmalogical inhibition affect the protein’s biological function in different ways, and therefore SIRT regulation needs to be cautiously manipulated concerning CNS diseases.

#### 4.2.3. Histone Ubiquitination in Neuroinflammation

**Figure 5:**
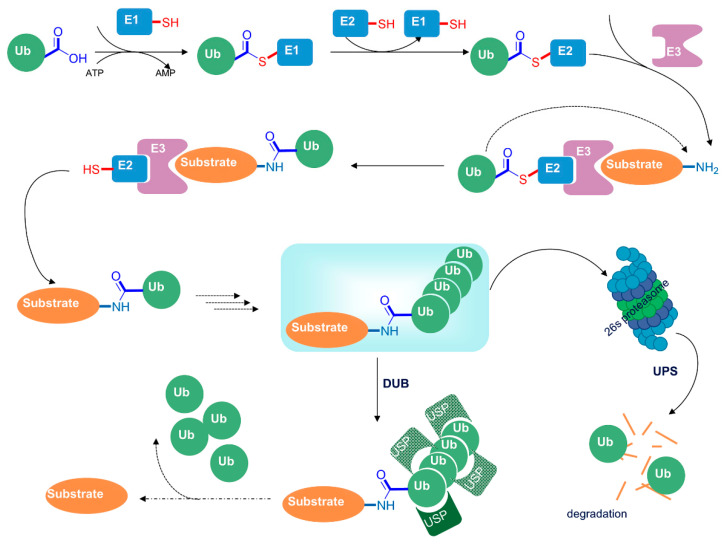
Schematic of cascade enzymatic reaction of ubiquitination. First, E1 enzymes activate ubiquitin in an ATP-dependent manner to form a thioester bond between the C-terminal glycine of ubiquitin and the cysteine site of E1. The activated ubiquitin subsequently forms a new thioester bond with the cysteine site of E2-conjugating enzyme. The resultant Ub-E2 conjugate cooperates with the E3 ligase to transfer the ubiquitin on the substrate to complete the ubiquitin labeling of the target protein, which is then degraded by the 26S proteasome. On the other hand, ubiquitin-specific protease (USP)-mediated deubiquitination enables the dissociation of ubiquitin from the substrate.

Ubiquitination is a multifaceted post-translational process that involves ubiquitin proteins enabling the classification of intracellular proteins and covalently binding to a lysine residue of the target protein. The ubiquitination process is a three-step cascade reaction that requires the participation of three functional enzymes including ubiquitin-activating enzymes E1, ubiquitin-conjugating enzyme E2 and ubiquitin ligase enzyme E3, each of which plays important role in ubiquitination and marks proteins for degradation by the proteasome [124,125,126]. Ubiquitin–protein conjugates are dynamic and unstable intermediates that can be dissociated by deubiquitinating enzymes (DUBs) to remove the ubiquitin molecules. By reversing the ubiquitinating process, DUBs are able to dispose inactive ubiquitin precursors and remove the ubiquitin from the conjugates to provide sufficient free ubiquitin within the cells [127,128] (Figure 5). Ubiquitin–proteasome pathway (UPP)-mediated proteolysis is the main molecular mechanism responsible for the normal function of the nervous system. The UPP regulates the degradation of the vast majority of misfolded proteins, and dysregulation of the UPP is linked to neurodegenerative diseases [129]. Additionally, ubiquitin-specific proteases (USPs) belong to the DUB family and have a vital role in deubiquitinating modification. Certain USPs are implicated in dozens of proinflammatory signaling pathways, such as NF-κB and TGF-β [130]. Given the importance of ubiquitination/deubiquitination in protein homeostasis, it is crucial for the regulation of a variety of signaling transductions and transcriptional regulations, as well as cellular processes and organismal homeostasis.

The transcriptional factor CCAAT/enhancer binding protein beta (c/EBPβ) is identified as a key regulator of pro-inflammatory genes in microglia, and it is overexpressed in AD [131,132,133]. Ndoja et al. found that microglia-specific deletion of COP1, an E3 ubiquitin ligase, led to a significant increase in activated microglia and astrocytes. And a deficiency of COP1 facilitates the elevation of c/EBPβ expression and the consequent activation of pro-inflammatory cytokines, thereby accelerating the neurodegeneration of AD [134]. In addition, the E3 ligase Pellino, expressed in a variety of nerve cells, is a key regulator of microglia-mediated autoimmune neuroinflammation, and it is engaged in the ubiquitination and degradation process of c/EBPβ. The depletion of Pelil can increase the expression of c/EBPβ and CD36, thereby promoting the phagocytic activity of microglia to further improve the clearance of Aβ [135,136]. Pleckstrin homology-like domain family A member 1 (PHLDA1) is an important modulator of microglia-mediated inflammation. A study by Han et al. demonstrated that PHLDA1 expression was elevated in LPS-induced microglia activation, while the absence of PHLDA1 could suppress the NF-κB signaling-related inflammatory response by interrupting K63-linked ubiquitination of the E3 ligase TRAF6. In MPTP-induced mice, PHLDA1 deficiency also showed marked activity in inhibiting neuroinflammatory reactions and ameliorating the behavioral impairment of mice, highlighting the potential of PHLDA1 in treating neurodegenerative diseases [137]. Cao et al. disclosed that the UPP contributes to receptor-interacting serine/threonine kinase 1 (RIPK1) degradation mediated by optineurin (OPTN) in microglial cells to inhibit the proinflammatory pathways of NF-κB and, thus, suppresses neuroinflammation [138]. F-box and WD-40-domain protein 11 (FBXW11) is a component of the SCF (Skp1-Cul1-F-box) E3 ubiquitin ligase complex [139,140]. A study conducted by Sun et al. showed FBXW11, a component of the SCF E3 ubiquitin ligase complex, was overexpressed in Aβ-stimulated microglia cells and the hippocampus of AD mice, and it enabled the activation of the ASK1/MAPKs/NF-κB inflammatory signaling pathway, thereby contributing to inflammation-induced AD pathology. Inhibition of FBXW11 provided neuroprotective effects and cognitive recovery of an AD model, providing a new insight into AD physiopathology and treatment options [141].

### 4.3. Histone Deacetylase and Histone Demethylase Inhibitors in Clinical Trials

**Figure 6:**
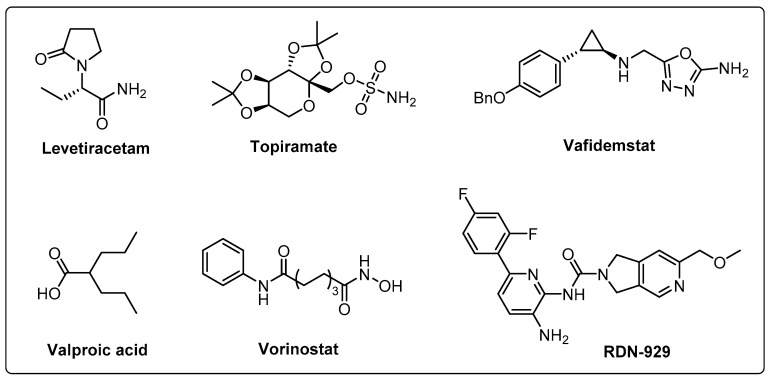
Chemical structures of HDACs and LSD1 inhibitors in clinical trials.

To date, numerous studies have elucidated the significance of epigenetic regulation in refractory human diseases like cancer and neurodegeneration. Therefore, targeting epigenetic modulators has emerged as a powerful and promising strategy to combat such diseases. However, most of the clinical development for epigenetic targets is attributed to cancer treatment, and less of them are linked to neurodegenerative diseases. We conducted a search on clinicaltrials.gov targeting the central nervous system, primarily focusing on AD. Our findings revealed that the agents currently under clinical assessment, which target histone and DNA modifiers, are predominantly repurposed drugs originally intended for other indications (Table 1), of which the chemical structures are shown in Figure 6.

Valproic acid, an antiepileptic drug, is a promising therapeutic approach in AD treatment. Unlike other HDAC inhibitors, Valproic acid selectively inhibits HDAC2 through a proteasomal degradation mechanism [142]. In preclinical studies, Valproic acid has demonstrated remarkable efficacy as an antiamyloid treatment in AD transgenic mouse models and notably stimulates neurogenesis of neural progenitor/stem cells in both in vitro and in vivo settings. Furthermore, Valproic acid shows significant amelioration of memory impairments and mitigation of neuroinflammation in key brain regions related to AD pathology, including the hippocampus and cortex [143,144,145]. These exciting findings highlight the therapeutic potential of Valproic acid in reducing inflammation and improving cognitive impairments associated with AD. Vorinostat is the first approved HDAC inhibitor for treating cutaneous T cell lymphoma. Preclinical studies showed that vorinostat can effectively reduce Aβ generation and neuritic plaque via inhibition of the GSK 3β-mediated γ-secretase cleavage of APP and thereby can mitigate memory deficits [143]. In addition, abnormal expression of H4K12 is closely related to age-associated memory deficits, and vorinostat treatment in aged mice can result in the elevation of H4K12 acetylation and the restoration of learing-relevant gene expression [113,146]. In addition, Topiramate and levetiracetam, as other antiepileptic drugs, have also demonstrated direct or indirect effects on HDACs. Preclinical studies showed that both drugs provide therapeutic benefits such as anti-inflammation, neuroprotection and correction of behavioral abnormalities and cognitive impairments [147,148]. RDN-929 is a brain penetrant and specific inhibitor of the HDAC-CoREST complex, which exhibits promise as a therapeutic agent for neurologic disorders characterized by impaired synaptic function. A phase I trial revealed that RDN-929 had a favorable safety, tolerability, and pharmacokinetic profile. Additionally, the observed enhancement of histone acetylation within peripheral blood mononuclear cells provided evidence of robust engagement with the intended target [149,150]. Vafidemstat (ORY-2001) is an orally bioactive dual inhibitor of LSD1 and MAO-B, with good blood–brain barrier penetration activity. Preclinical studies showed that ORY-2001 is able to alleviate cognitive impairments in an SAMP8 mouse model, reduce neuroinflammatory factor generation, and rectify deregulated genes associated with cognitive function and neuroplasticity [151]. ORY-2001 also showed therapeutic efficacy in multiple degenerative models by reducing neuroinflammation and modulating glial activity, thereby exhibiting neuroprotective effects.

## 5. Conclusions and Prospects

AD is a multifaceted disease caused by the joint action of genetic and environmental factors, and the signal hypothesis of linear causality based on Aβ and tau can not fully address the pathogenesis of AD. Currently, there is a paucity of effective therapeutic options for AD, while the present clinical treatment merely provides limited symptomatic relief. Growing evidence has shown that neuroinflammation plays an essential role in driving the onset and progression of neurodegenerative diseases, and thus has received great attention as a potential therapeutic target for AD treatment. A wide array of findings has revealed that the molecular alternations in inflammatory signaling are governed by epigenetic regulation. Epigenetic mechanisms are tightly involved in gene expression and molecular signaling pathways, thereby participating in the coordination and adaptation of the body to external environmental changes and body aging, as well as in the occurrence and development of various diseases.

Epigenetic modifications have an important role in AD pathology and cognitive function through the involvement of APP metabolism, Aβ formation, tau protein phosphorylation, oxidative stress response, cell apoptosis, and inflammatory responses. Early diagnosis and intervention can help prevent and treat AD; however, there has been a lack of effective detection indexes so far. Changes in inflammation-related epigenetic regulations are capable of providing effective indication for AD diagnosis. Dysregulated epigenetic mechanisms such as histone methylation and acetylation are closely associated with inflammation-induced learning and memory deficiencies. However, understanding of the exact molecular regulatory mechanism still remains limited. Additionally, due to the lack of specificity of gene regulation, many epigenetic interventions may affect other molecular mechanisms and signaling transduction. Additionally, epigenetic modulators usually regulate gene expression and the inflammatory signaling cascade, mainly in the form of protein complexes that may be affected by different intervention approaches [152]. For instance, specific genetic depletion of target modulators may elicit dissociation of the protein complex and thereby release the cofactors to activate multiple signaling cascades, resulting in unexpected phenotypes. In contrast, pharmacological intervention with small molecules mainly interferes with the catalytic activity of the target protein without affecting its expression, but the outcomes are highly dependent on the targeting specificity of small molecules. Although the role of epigenetic regulations in inflammation regarding neurodegenerative diseases has been extensively explored, there currently remains a paucity of reliable biological indicators for AD diagnosis and treatment. Further studies to insightfully elucidate the interplay between the epigenetic control and neuroinflammation may help to determine the therapeutically beneficial markers and provide additional treatment strategies for patients with AD. In addition, the selectivity of current HDAC inhibitors remains limited as their effects on the different subtypes of HDACs implicated in AD development are not fully elucidated. To advance our understanding of the underlying mechanisms related to the impairment of memory and learning in AD, it is imperative to determine the specific subtypes of HDAC family members associated with the disease’s pathology. 

## Figures and Tables

**Table 1:** Histone deacetylase and demethylase inhibitors in AD in progress.

Drug	Epigenetic Target	Phase	Trial Identifier	Outcomes in (Pre)Clinical Studies
Levetiracetam	HDACs	2	NCT04004702	Reduce proinflammatory factors, decrease cell death and oxidative stress, mitigate cognitive impairment.
		2	NCT03875638	
		2	NCT02002819	
		2	NCT03461861	
		4	NCT05969054	
		2	NCT03489044	
		3	NCT05986721	
Topiramate	HDACs	1	NCT02884050	Inhibit microglia activation, activate Akt and AMPK, facilitate Aβ transport and clearance.
Valproic acid	HDACs	3	NCT00071721	Reduce inflammation, inhibit Aβ deposition, improve cognitive impairments in APP23 and APPswe/PS1ΔE9 AD mice.
		1	NCT01729598	
		2	NCT00088387	
Vorinostat	HDACs	1	NCT03056495	Regulate synaptic plasticity and improve cognitive function, restore gamma oscillation deficits in PSAPP mice, reduce inflammation.
RDN-929	HDAC-CoREST	1	NCT03668314	Reactivate neuronal gene expression, strengthen synaptic function, promote synapse formation.
		1	NCT03963973	
Vafidemstat	LSD1, MAO-B	2	NCT03867253	Improve learning and mitigate memory deficit in SAMP8 model and decrease inflammation in the hippocampus.

## Data Availability

No new data were created or analyzed in this study. Data sharing is not applicable to this article.
